# Validation of a functional human AD model with four AD therapeutics utilizing patterned iPSC-derived cortical neurons integrated with microelectrode arrays

**DOI:** 10.21203/rs.3.rs-4313679/v1

**Published:** 2024-05-20

**Authors:** Julbert Caneus, Kaveena Autar, Nesar Akanda, Marcella Grillo, Chris Long, Max Jackson, Sarah Lindquist, Xiufang Guo, Dave Morgan, James J Hickman

**Affiliations:** University of Central Florida; University of Central Florida; University of Central Florida; University of Central Florida; Hesperos Inc; Hesperos Inc; Hesperos Inc; University of Central Florida; Michigan State University; University of Central Florida

**Keywords:** Alzheimer’s Disease, long-term potentiation, iPSCs, therapeutics, in vitro microelectrode array

## Abstract

Preclinical methods are needed for screening potential Alzheimer’s disease (AD) therapeutics that recapitulate phenotypes found in the Mild Cognitive Impairment (MCI) stage or even before this stage of the disease. This would require a phenotypic system that reproduces cognitive deficits without significant neuronal cell death to mimic the clinical manifestations of AD during these stages. A potential functional parameter to be monitored is long-term potentiation (LTP), which is a correlate of learning and memory, that would be one of the first functions effected by AD onset. Mature human iPSC-derived cortical neurons and primary astrocytes were co-cultured on microelectrode arrays (MEA) where surface chemistry was utilized to create circuit patterns connecting two adjacent electrodes to model LTP function. LTP maintenance was significantly reduced in the presence of Amyloid-Beta 42 (Aβ42) oligomers compared to the controls, however, co-treatment with AD therapeutics (Donepezil, Memantine, Rolipram and Saracatinib) corrected Aβ42 induced LTP impairment. The results presented here illustrate the significance of the system as a validated platform that can be utilized to model and study MCI AD pathology, and potentially for the pre-MCI phase before the occurrence of significant cell death. It also has the potential to become an ideal platform for high content therapeutic screening for other neurodegenerative diseases.

## INTRODUCTION

The majority of prospective new drug candidates show great promise in preclinical studies but fail in clinical trials [1–5]. This disparity can be attributed in part to the dearth of relevant experimental models used during preclinical studies for assessing drug safety and efficacy,

The main pathological features of AD include the formation of extracellular amyloid plaque deposits, intracellular neurofibrillary tangles (NFTs) formed by hyperphosphorylation of tau protein, and a large degree of neuronal cell death and synaptic changes [14]. In addition to Aβ toxicity [15] and tau protein associated toxicity [16, 17], other factors that contribute to dementia risk include oxidative stress [18], inflammation [19], cholinergic neuronal damage [20] and neurovascular changes [21], based on pathology and genetic risk factors [22]. According to the amyloid hypothesis, the initiating factor in AD is the accumulation and aggregation of the Aβ peptide [23]. Evidence indicates that soluble oligomers of Aβ, including dimers, trimers, dodecamers and spherical aggregates of 6 nm Aβ-derived diffusible ligands(ADDLs) and 12 nm amylospheroids (ASPD) [24] have significantly higher toxicity in vitro than either the monomeric or the larger fibrillar aggregates [25–31]. Moreover, soluble oligomers of Aβ have profound physiological effects at low concentrations without signs of plaque formation [32–36].

In spite of the significant progress in elucidating the biological mechanisms of AD [37, 38], no practical treatments exist which prevents or significantly delays its progression. This is especially problematic in that by the time symptoms are evident during the mild cognitive impairment (MCI) stage, significant brain neuropathology and even cell death has already occurred [39–41]. Yet, most clinical trials have focused on treating advanced stages of the disease [42, 43]. It is now becoming apparent that treatment windows, and thus clinical trials, must shift to the MCI, or better yet the pre-MCI stage, to be effective, however there are few diagnostics, if any, to predict who will develop AD at this stage of the disease [44–46].

Drug discovery can be divided into two general classes – target-based approaches and phenotype-based approaches [47]. Target-based drug discovery has been the most common approach since it is more amenable to the development of high throughput screening (HTS) assays. The advantage of target-based biochemical/genetic approaches is that large numbers (thousands to millions) of rationally designed compounds can be rapidly screened with today’s automated robotic systems in a cost-effective manner. Disadvantages are that multiple positive hits (possibly in the hundreds) targeting single biochemical/genetic pathways are normally identified and no information is obtained from this type of screen on the eventual physiological effect of the compounds at the tissue level. Phenotypic-based drug screens utilize single cells, nematode worms, zebrafish and tissue engineered organs mimics as a primary or secondary in vitro screen for better prediction of which compounds are the best candidates to move into small animal studies [47–51]. These are normally termed High Content Screens (HCS) and provide a more ‘holistic’ analysis of a compound’s effect on multiple pathways. While these HCS assays are more labor-intensive and costly than HTS, they are less expensive and more rapid than small animal studies. While HCS assays can be used to reduce the dozens to hundreds of HTS positive hits to a few attractive targets to take into small animal studies, currently these assays are not used as substitutes for whole animal studies but serve as a bridge between HTS single target assays and in vivo studies. However, recent work provided support for testing C1s inhibition in a clinical trial of an autoimmune demyelinating neuropathy, CIDP (NCT04658472), and suggests great potential for microphysiological systems use for translational research leading to IND generation [52]. This also suggests MPS systems can be used to generate efficacy data for repurposing drugs or drug candidates without the use of animal data.

In this report we simulated AD pathology in our in vitro system by application of toxic forms of Aβ oligomers and evaluated the functional deficits using circuits of cortical neurons, and identified AD-relevant functional deficits. Previously, we demonstrated that Aβ42 and Tau oligomer application to a cortical neuron MEA platform resulted in pronounced deficits in stimulus-induced neuronal activity from LTP maintenance [53]. To evaluate the system’s capacity to assess the effects of AD therapeutics and its potential as a prospective tool in drug development, the systems were treated with Aβ42 oligomers and different classes of AD-based drugs, which included Donepezil, Memantine, Rolipram and Saracatinib. These are all either currently approved or in the case of Saracatinib, previously approved but is now withdrawn.

Following Aβ42 and drug dosing, the functional activity (i.e., LTP maintenance) of cortical neurons was examined. The results revealed a pronounced decrease in cell activity within 1hr of Aβ42 dosing, which was blocked by co-administration of the Aβ42 oligomers and AD drugs simultaneously. These results underline the significance and potential of the LTP phenotypic cognitive functional assay system as an applicable tool in the drug development process, which can be employed to quickly analyze promising therapeutic compounds as another metric for efficacy testing of AD therapeutics, but with only milligrams of a drug candidate. It also provides a tool that can be utilized at the pre-MCI or before phase of AD as the deficits in LTP occur without neuronal cell death. Moreover, this system provides a foundation for the development of higher order, more complex models, which can be used to not only study the drug effects, but also the mechanism of action of potential therapeutics as well.

## RESULTS

### 2.1 Analysis of iPSC-derived cortical neurons for the expression of AD drug targets

Currently, Donepezil, Memantine, and Rolipram are all FDA-approved drugs for the treatment of AD. Donepezil targets and binds to acetylcholinesterase enzyme (AChE), subsequently preventing it from catalyzing the hydrolysis of the neurotransmitter acetylcholine (ACh).

**Figure 1:**
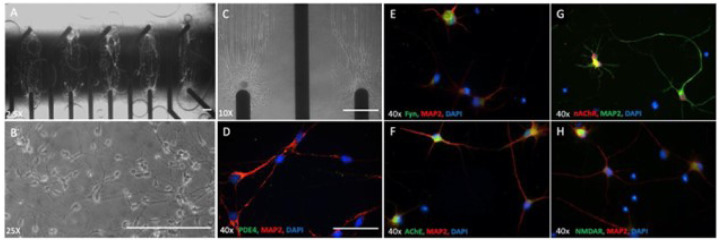
Expression of the molecular targets of AD drugs in hiPSC-derived cortical neurons. Phase images of hiPSC-derived cortical neurons distributed on patterns aligned with MEA electrodes(**A-C**), **scale bar = 100um.**.(**D-H**) Immunocytochemistry of hiPSC-derived cortical neurons utilizing antibodies specific to target proteins relevant for AD drugs revealed the cells expressed all markers evaluated, **scale bar = 50um.**.

Memantine binds to the NMDA receptor and blocks the channel from opening and sequentially cell excitotoxicity by glutamate. Rolipram inhibits the excessive degradation of cyclic AMP (cAMP) in the presence of Aβ42 by inhibiting the enzyme phosphodiesterase type 4 (PDE4) that catalyzes its breakdown. To determine whether the drugs targets (e.g., Fyn, NMDAR, AChE, nAChR and PDE4) are expressed in the cortical neurons, the cells were fixed and stained with specific antibodies for each marker followed by microscopy analysis. Figure 1 illustrates neuronal morphology (Fig. 1B) and alignment on patterned MEAs (Figs. 1A–1C). Additionally, the cells were stained positive for all the markers, including PDE4 (Fig. 1D), Fyn (Fig. 1E), AChE (Fig. 1F), nAChR (Fig. 1G) and NMDAR (Fig. 1H),

### 2.2 Analysis of Donepizil treatment on Aβ oligomer induced electrophysiological dysfunction in hiPSC-derived cortical neurons

A reduction in acetylcholine neurotransmitters in the brain, due in part to its breakdown and degradation by the actetylchonlinesterase enzyme, has been implicated in the development and/or progression of Alzheimer’s disease pathogenesis [67, 68]. Donepezil is an acetylcholinesterase inhibitor therapeutic and has received FDA approval for the treatment of AD [68]. To evaluate the patterned cortical MEA system’s effectiveness in assessing drug effects by changes in LTP and its potential as a prospective tool for drug screening.

**Figure 2:**
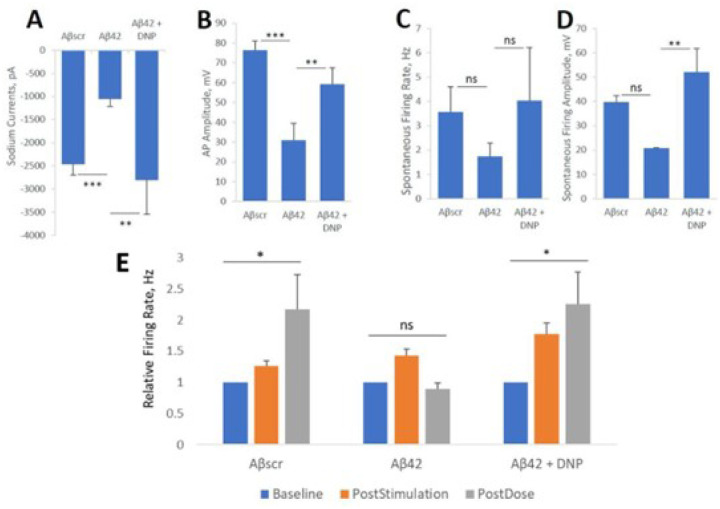
Amyloid beta42 oligomers induced neurotoxicity of hiPSC-derived cortical neurons is inhibited by Donepezil. (**A-D**) Patch clamp electrophysiology recordings from the hiPSC-derived cortical neurons indicated the blocking of Aβ42-induced deficits by co-treatment with Donepezil (1 μM) for 24 hours, as demonstrated for the readouts of sodium currents (**A**), action potential (AP) amplitude (**B**), spontaneous firing rate (**C**) and amplitude (**D**). (**E**) Analysis of cell function on patterned cortical MEA systems revealed a stimulus-induced increase in cell activity (i.e., firing frequency) was maintained in control samples dosed with Aβscr (5 μM), but was completely abolished within 1h of Aβ42 oligomers dosing. However, this Aβ42-induced abolishment was blocked by co-treatment with donepezil (DNP, 1 μM) (E). Statistical analysis was computed using either Student t-test or One-Way ANOVA with Tukey’s test and Alpha (0.05) is significance. (N ≥ 16), *p ≤ 0.05, **p ≤ 0.01, ***p ≤ 0.001.

To assess the capacity of the system for evaluating drug effects, in paralell experiments, iPSC- derived cortical neuronal cells were plated on both coverslips and on patterned MEAs and maintained for 28–35 days in culture prior to testing. Patch clamp recordings were preceeded by a 24hr acute dose evaluating individual neuronal cell dynamics according to our previous protocol [53], while MEA experiments investigated 1hr acute responses on LTP maintenance of neuronal population dynamics and synaptic connectivity according to Caneus et al [53]. On the day of testing, at 24h post-dosed, neuronal function was recorded and analyzed by patch clamp as shown in Fig. 2. The administration of Aβ42 oligomers to the cultures resulted in a sharp decrease in cell firing potential. Notably, a significant decrease in both sodium and potassium currents was observed in cells exposed to Aβ42 oligomers compared to Aβscr oligomer-treated cells (Fig. 2A). Additionally, the data revealed a pronounced reduction and sometimes a complete lack of activity of both action potentials (AP) and spontaneous firing in the cells dosed with Aβ42 oligomers relative to samples dosed with Aβscr oligomers. However, co-treatment with Donepezil (1μM) indicated a clear protection on neuronal function from the effects of Aβ42 oligomers, with most of the parameters analyzed demonstrating statistically significant differences (Figs. 2B–2D).

Similarly, in the hiPSC-derived cortical neuronal MEA system, the increase in neural activity induced by HFS was able to be maintained at 1 hr testing in the Aβscr control group, abloished in the Aβ42 group, but was recovered in the Donepezil group, which demonstrating an LTP amplitude comparable to the Aβscr control group (Fig. 2E). After testing, each MEA system was treated with lidocane to establish that the observed signals were bilogical in nature and not electronic noise. This is necesaary as electonic noise can resemble AP wave forms depending on the filters employed and the algorithms utilized to analyze the data. Therefore, the patch clamp and MEA data confirmed the therapeutic effect of Donepezil by blocking the Aβ42- induced neurotoxic effects and preserving the cell functionality.

**Figure 3:**
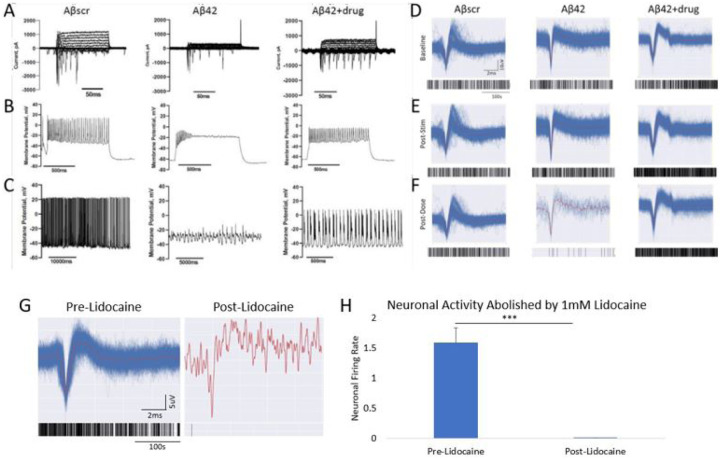
Correlation of the effects of an AD approved drug against Aβ42 oligomers induced neurotoxicity in cortical neurons utilizing two separate functional measurements. **(A-C**) Patch clamp electrophysiology recordings indicated a marked reduction in sodium currents in hiPSC-derived cortical neurons following 5 μM Aβ42 oligomer application at 24 hrs post- treatment (**A**). Additionally, a significant decrease in both induced action potential firing under depolarization (**B**) and spontaneous firing peak amplitudes (**C**) was detected in cells treated with Aβ42 relative to cells dosed with amyloid beta scrambled (Aβscr), but the decrease was ameliorated by co-treatment with AD drugs. (**D-F**) Similar observations for activity in the parallel cortical MEA systems were observed. Following establishment of baseline activity levels (**D**), the induced LTP activity was maintained at 1hr post dosing (**E**), and in samples treated with Aβscr (5 μM) no change was observed, a sharp decrease was observed in the samples dosed with Aβ42 oligomers alone within 1hr of treatment and co-treatment of the Aβ42 oligomers and Donepizil rescued the loss of activity (**F**). (**G**) Neuronal activity traces and accompanying raster plots illustrating the abolishment of biological activity through blocking of Na^+^ channels with the addition of 1mM Lidocaine. (**H**) Graph quantification of **G** highlighting the abolishment of neuronal signals following lidocaine addition. Statistical analysis was determined via Student’s T-test. *p < 0.05, **p < 0.01, ***p < 0.001.

Neuronal activity analysis was confirmed via the addition of 1mM Lidocaine upon completion of LTP testing. After Lidocaine addition, neuronal signals were completely or near-completely abolished (Fig. 3G). The abolishment of neuronal signals within the drug experiments was found to be statitically significant (Fig. 3H), further confirming that all neuronal signals used within the analysis for this study were biological.

### 2.3 hiPSC-derived Cortical neurons retained normal electrical function following co- treatment with (Aβ42) oligomers and Saracatinib, Memantine or Rolipram.

#### Saracatinib

**Figure 4:**
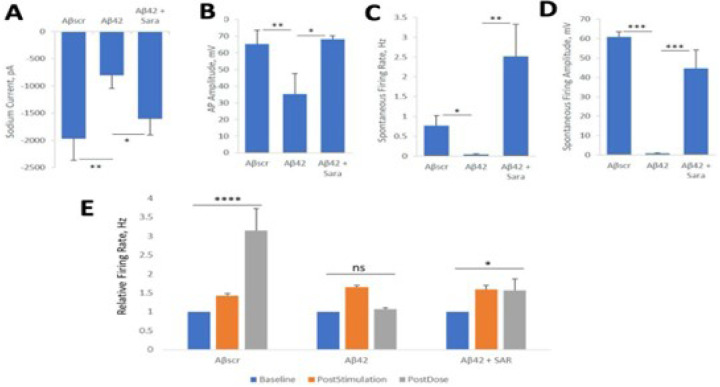
Saracatinib blocks amyloid beta42 oligomers toxic effects on hiPSC-derived cortical neurons. (**A-D**) Patch clamp electrophysiology recordings from the hiPSC-derived cortical neurons showed the blocking of the Aβ42-induced defects by co-treatment with Saracatinib (10 nM) for 24 hours, as demonstrated for the readouts of Sodium currents (**A**), AP amplitude (**B**), spontaneous firing rate (**C**) and amplitude (**D**). (**E**) Analysis of cell function on cortical-MEA systems. A stimulus-induced increase in cell activity (i.e., firing frequency) was maintained in control samples dosed with Aβscr (5 μM), but was completely abolished within 1h of Aβ42 oligomers dosing. However, this Aβ42-induced abolishment was blocked by co-treatment with Saracatinib (10 nM). Statistical analysis was computed using either Student t-test or One-Way ANOVA with Tukey’s test where applicable. Alpha (0.05) is significance. (N ≥ 25),*p ≤ 0.05, **p ≤ 0.01, ***p ≤ 0.001

To further investigate the neuroprotective effects of AD drugs against Aβ42 oligomer neurotoxicity cells were plated on patterned coverslips and treated with Aβ oligomers The neuronal functional activity was measured intracellularly by whole cell patch electrophysiology. Similar as the results in above sections, the addition of Aβ42 oligomers to the cortical cultures resulted in prominent cell dysfunction within 24h post-dosing, and co-treatment with saracatinib resulted in a blocking of the Aβ42 -induced dysfunction in all the with or without the saracatinib treatment in parallel experiments with Aβscr as in the control. parameters analysed including reduced currents (Fig. 4A), induced potential amplitude (Fig. 4B) and spontaneous firing (Fig. 4C–D). To investigate whether Saracatinib offers some protective effects against Aβ42-induced impairment in LTP, cortical-HoAC systems were established and co-treated with saracatinib together with Aβ42 oligomers. The cells were dosed with either Aβscr or Aβ42 oligomers with or without Saracatinib (10 nM) immediately after HFS. As indicated in Fig. 4E, the cells exhibited a significant increase in firing frequency from baseline activity following stimulation. The induced activity was significantly diminished in the samples dosed with Aβ42 oligomers relative to Aβscr-treated samples at 1h post-dosed. However, simultaneous administration of saracatinib together with Aβ42 oligomers inhibited the Aβ42-induced deficits in cell firing and preserved the induced cell activity in the Aβ42- Saracatinib treated samples compared to Aβ42 only treated samples (Fig. 4E).

Figure 4. Saracatinib blocks amyloid beta42 oligomers toxic effects on hiPSC-derived cortical neurons.

#### Memantine

**Figure 5:**
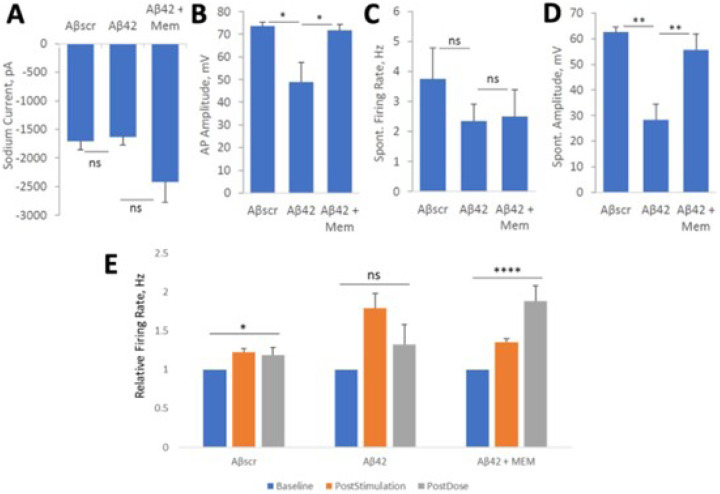
Memantine suppresses amyloid beta42 oligomers neurotoxic effects on hiPSC-derived cortical neurons. (**A-D**) Patch clamp electrophysiology recordings from the hiPSC-derived cortical neurons show the blocking of Aβ42-induced defects by co-treatment with Memantine (5 μM) for 24 hours, as demonstrated for the readouts of Sodium currents (A), AP amplitude (B), spontaneous firing rate (C) and amplitude (D). (**E**) Analysis of cell function on cortical-MEA systems, indicated a stimulus-induced increase in cell activity was maintained in control samples dosed with Aβscr (5 μM), but was completely abolished within 1h of Aβ42 oligomers dosing. However, this Aβ42-induced abolishment was blocked by co-treatment with memantine (5 μM). Statistical analysis was computed using student t-test or One-Way ANOVA with Tukey’s test and Alpha (0.05) is significance. (N≥25), *p ≤ 0.05, **p ≤ 0.01, ***p ≤ 0.001,****p ≤ 0.0001.

To evaluate the effects of Memantine on Aβ42-induced cell defects, the cell were treated for 24 hrs and analyzed by whole-cell patch electrophysiology and MEA analysis. Based on the results demonstrated in Fig. 5, the application of Aβ42 oligomers abolished most of the neuronal activity, but the co-treatment with Memantine counteracted the effect of Aβ42 in all the parameters analyzed including cell currents (Fig. 5A), induced potential amplitude (Fig. 5B) and spontaneous firing and amplitude (Fig. 5C–D), in comparison to samples treated with Aβscr or co-treated with Aβ42-memantine. To assess the protective effects of Memantine on cortical neuronal MEAs they were treated and analyzed as described above. The results demonstrated a marked increase in cell firing frequency from baseline recording following stimulation in all the samples (Fig. 5E). Whereas the samples treated with Aβscr oligomers continued to maintain this activity at 1h after dosing and the samples treated with Aβ42 oligomers demonstrated a prominent decrease in cell activity from post-stimulation recording. Memantine demonstrated an ability to inhibit the Aβ42-induced 1 hr LTP deficits (Fig. 5E). In all, these findings indicate that Memantine blocks Aβ42-induced neurotoxicity.

#### Rolipram

**Figure 6:**
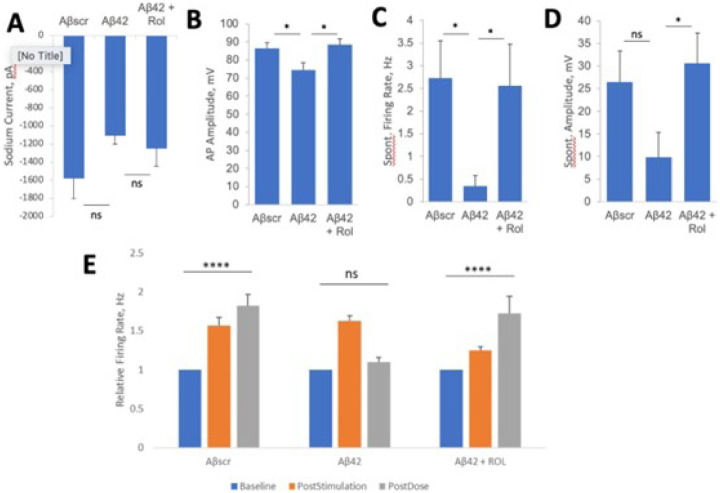
Rolipram suppresses Aβ42 oligomers neurotoxic effects on hiPSC-derived cortical neurons. (**A-D**) Patch clamp electrophysiology recordings from the hiPSC-derived cortical neurons showed the blocking of the Aβ42-induced defects by co-treatment with rolipram (1 μM) for 24 hours, as demonstrated for the readouts of Sodium currents (A), AP amplitude (**B**), spontaneous firing rate (**C**) and amplitude (**D**). (**E**) Analysis of cell function on cortical-MEA systems showed a stimulus-induced increase in cell activity was maintained in control samples dosed with Aβscr (5 μM), but was completely abolished within 1h of Aβ42 oligomers dosing. This Aβ42-induced abolishment was blocked by co-treatment with rolipram (1 μM). Statistical analysis was computed using student t-test or One-Way ANOVA with Tukey’s test and Alpha (0.05) is significance. (N ≥ 18), *p ≤ 0.05, **p ≤ 0.01, ***p ≤ 0.001, **** p ≤ 0.0001.

Whole-cell patch clamp results revealed the potential of rolipram to prevent neurotoxic effects of Aβ42. Though not significant, the co-administration of rolipram along with Aβ42 oligomers for 24 hrs blocked sodium current deficits observed in the Aβ42 only condition (Fig. 6A). Rolipram did, however, significantly block deficits in spontaneous firing frequency and ampltitude (Fig. 6B–D). [69]. To evaluate the neuroprotective abilities of rolipram against Aβ42 induced toxicity in LTP, cortical-MEA systems were stimulated and analyzed as desribed above. Rolipram was observed to have neuroprotective effects, as co-administration of rolipram with Aβ42 oligomers caused a highly significant increase in event rate at 1h following LTP induction, comparable to the persistent LTP observed in the Aβscr treated condition. In contrast, the systems dosed with only Aβ42 oligomers had a significant decrease in activity at 1hr post-stimulation (Fig. 6E).Overall, rolipram was observed to inhibit the neurotoxic effects of Aβ42 oligo

## DISCUSSION

The drug discovery and development process has been an area of great interest to scientists both in academia and pharmaceutical companies [70–72]. Yet, despite the significantly large investments being dedicated towards this process [73–76], the procedures remain stagnant and inefficient, (with less than 15% of all promising new therapeutic compounds receiving marketing approval [3, 7]). This paucity is even more striking for neurological disorders [77, 78], for instance, it had been more than 18 years since the last drug was approved for AD treatment, before the recent controversial approval of Aducanumab by the FDA [79–82]. Nevertheless, during that same period, there have been many candidate drugs that showed great promises in preclinical studies but failed in clinical studies [77, 83, 84]. While there may be many factors that contribute to the high attrition rate of investigative drugs, preclinical models, especially animal models, are believed to be the predominant reason, where efficacy (52%) or safety (24%) accounts for the majority of drug failures in clinical trials [85]. Historically, the drug discovery process has relied largely on animal models for safety and efficacy research in order to obtain preclinical evaluation of promising new therapeutic compounds [6, 86, 87]. However, genetic differences between animals and humans is regarded as one of the main factors contributing to the very high attrition rate in human studies [1, 6, 11]. The lack of compatible models to study human diseases has greatly impeded the drug development process.

The recent advances in the development and differentiation of iPSCs into mature cells now provide a new source and endless possibilities to create more relevant models [88]. Today, based on the relative ease with which different types of human cells (especially neuronal cells, which are normally inaccessible for scientific research prior to death) can be generated from iPSC cells, many researchers are re-evaluating their research approaches and putting more emphasis towards developing more compatible, iPSC-derived cells based in vitro models to study human diseases and for drug development [89]. Previously, we generated a human-based system using cortical neurons derived from iPSCs to study aspects of AD pathophysiology without neuronal death [53]. Here, using the four FDA-approved, AD-related drugs, the system’s capacity was investigated as a potential platform for drug screening for AD, or more specifically for mild cognitive impairment (MCI). This is important as there are few, if any, screens for the MCI, or pre-MCI, stages of the disease, which this system can address. As indicated in the results above, a significant increase in cell firing frequency from baseline activity was detected in the system following stimulation using a HFS protocol. This HFS-induced increase was maintained at least for 1 hour and is defined as LTP. Which is a correlate for learning and memory [53]. The stimulus-induced change in cell activity was subsequently abolished by Aβ42 oligomer dosing while preserved in the Aβscr group, but without cell death. Both Donepezil and Memantine have been approved by the FDA as separate or a combined (Namzaric) drug for AD treatment, [90]. Both drugs, as well as Rolipram and Saracatinib, were evaluated individually in this cortical neuron AD platform. The Aβ42 oligomers neurotoxic effect was inhibited in the presence of the AD-related drugs, demonstrating the system proficiency to capture Aβ42 induced functional deficits and their rescue by the tested drug. The reason for utilizing different treatment duration for these two assays is because of the different focus of these two assays. Patch clamp recordings evaluates individual neuron electrophysiological activity, while MEA experiments investigated the LTP maintenance in the neural populations of dynamic circuit activity and active synaptic interactions. Also, our previous work already demonstrated the validity of these two protocols in inducing and evaluating AD-relevant phenotype in these two assays [53].

While more studies are needed to expand on the findings presented here and to further validate the effectiveness and applicability of the system as a platform for drug screening, the findings, nonetheless, provide evidence to support the utility of the system as a more relevant human-based in vitro system to model human disease at the preclinical stage, especially for the MCI stage of the disease. Furthermore, these human-on-a-chip systems are potentially significant for a number of important reasons: 1) they provide a human-based in vitro model to study disease pathophysiology (i.e., mechanisms and pathways) in vitro, 2) they provide a simple, reproduceable and economically effective tool for drug screening (e.g., toxicity and efficacy), 3) they provide a foundation for designing and creating more complex systems which can be used to study the mechanism of drug reaction, mode of transport and to screen libraries of already approved drugs for drug repurposing, 4) they are scalable, and can be used for high-content drug testing, and 5) they can be used in the application of personalized medicine. Data generated with these models can also provide support for repurposing existing drugs as indicated by a previous study for testing C1s inhibition in a clinical trial of the autoimmune demyelinating neuropathy, CIDP (NCT04658472), and suggests great potential for microphysiological systems use for translational research leading to IND generation [52].

Despite the connection of Aβ accumulation to AD, it is now accepted that the amyloid plaques are not sufficient to cause the symptoms of AD. It appears that in addition to Aβ, there is a requirement for the spread of tau pathology throughout the brain regions critical for learning and memory before the disease becomes manifest. Experimental studies in transgenic mice suggest that addition of Aβ to mouse models of tauopathy can exacerbate the amount of tau deposition and accelerate the development of the tau phenotype [91, 92]. Importantly, while murine models of amyloid pathology generally fail to result in major neuronal loss [93, 94], murine tau models have extensive neuronal loss and brain atrophy [95, 96]. It is also recognized that the amount of tau pathology is more closely associated with the extent of cognitive decline in older adults and AD cases than the amount of amyloid pathology [97]. The dual impacts of amyloid and tau on neural function cause us to evaluate each of these as possible initiators of spinal and PNS changes found in AD. In light of tau’s significant effects on AD future studies will investigate this important variable in AD pathology.

## CONCLUSIONS

This study sought to investigate the ability of a human-on-a-chip CNS model to validate the proficiency of current AD drugs in the prevention of cognitive dysfunction in the presence of AB42 oligomers. This study employed the utilization of an in vitro, human-based system to recapitulate clinical findings to enable further validation of HoaC systems for pre-clinical drug testing. The culturing of neuronal populations on microelectrode arrays enabled an emphasis on synaptic plasticity, and LTP could therefore quantitatively be used to investigate the efficiency of the AD therapeutic treatments to prevent the inhibition of LTP.

Throughout this study, it was shown that each of the four drugs used: Donepezil, Memantine, Saracatinib, and Rolipram, were able to successfully block AB42-induced cognitive deficits via whole-cell patch clamp and MEA recordings. Both readouts concluded that neuronal function and synaptic plasticity in the form of persistent LTP were preserved in the presence of the AD drugs, respectively.

This model can be further adapted to investigate rescue effects of AD therapeutic treatments, as well as cognitive deficits arising from other mechanisms relating to the onset of AD, such as the presence of phosphorylated Tau. This study has illustrated how this serum-free, in vitro system can recapitulate the preservation of neuronal function following treatment akin to that observed in clinical settings. Thus, its utilization can enable smaller-scale reproducibility of pre-clinical drug effects in a human-derived system.

## METHODS (Study Design)

This project aimed to validate an hiPSC-derived cortical neuron system for monitoring AD- relevant functional deficits to enable the evaluation of the effectiveness of AD therapeutics. Two functional assays were employed: patch clamp analysis on neurons cultured on coverslips and LTP analysis of neural circuits cultured on patterned MEAs. To evaluate the response of Aβ oligomer treatment, cells on coverslips were treated with the Aβ42 oligomers, control scrambled Aβ oligomers (Aβscr), or Aβ42 oligomers plus AD therapeutics for 24hr and then subjected to patch clamp analysis. Those on MEAs were recorded for baseline activity, high frequency stimulation for LTP induction, then treated with Aβ42 oligomers, Aβscr, or Aβ42 oligomers plus AD therapeutics for 1 hr and the LTP activity was measured. For patch clamp electrophysiology, the parameters analyzed were: amplitude of the sodium current, amplitude of the Action Potentials (AP), number of spontaneous firrings with a 30 sec duration, and amplitude of spontaneous firing. For the MEA assay, the parameter analyzed was the neural activity at 1 hr post LTP induction normalized by baseline activity. For each drug evaluated, there were three experimental groups: cells treated with Aβscr as the control, those treated with Aβ42 oligomers as the AD pathological group, and those treated with Aβ42 oligomers plus AD drug as the therapeutic treated group. The AD deficit was evaluated by comparing between the Aβ42 group and the control, and the drug’s effect was evaluated by comparing between the Aβ42 + drug group with the Aβ42 group. The number of systems tested was determined statistically so as to detect a significant difference between the Aβ42 group and the control, with a type I error rate (α) of 0.05 using a Dunnetts’s multiple-comparisons test to the control condition.

### 3.1 Neural Cells:

Human cortical neurons were used to assess the effects of multiple classes of AD therapeutics on Aβ oligomer induced neuronal cell dysfunction. The cells were derived from human iPSCs from healthy individuals and were either purchased from Cellular Dynamics International (CDI, iCell GlutaNeurons, Cat. #: C1033, Madison,WI) or defferentiated directly in our lab as described previously [53–55]. Human primary astrocytes were purchased from ScienCell (Cat. # 1800).

### 3.2 Surface chemistry:

Neuronal surface patterning on custom MEA chips was prepared using a surface coating protocol of polyethylene glycol (PEG), followed by laser ablation and backfill coating with DETA (N-1(3-[trimethoxysilyl]propyl)-diethylenetriamine) as detailed in Wilson et al ([56]). Subsequently, neuronal attachment was further encouraged with a protein adsoprtion coating of poly-L-ornithine (PLO) and laminin, which consisted of a 1hr RT incubation of 0.01%

PLO solution, followed by 3X rinses of 1X PBS before incubating overnight at 4°C with 10 μg/ml laminin solution ([55]). The laminin solution was removed prior to cell seeding.

### 3.3 Cell Culture:

The cells were seeded on the patterned MEA surfaces and on coverslips. A monoculture of cortical neurons were plated at a density of 150 cells/mm^2^ on coverslips for immunostaining (ICC) analysis and patch-clamp electrophysiology as decribed previously [35, 53]. For the MEA cultures, a co-culture of cortical neurons and astrocytes were seeded directly onto patterned MEAs to promote cell adhesion of separated cell-clusters on the individual electrodes, as well as the formation of synaptic connections between two adjacent electrodes. The neuronal cells were plated at a density of 500 cells/mm^2^ and the astrocytes at 250 cells/mm^2^. The cells were were first plated in the manufacture’s (CDI) recommended medium for the first 24h before they were switched to a serum-free medium. The cells were maintained in culture in the serum-free medium for 28–35 days prior to dosing and testing [53].

### 3.4 Aβ Oligomers Preparation:

The Aβ oligomers were prepared using peptides from rPeptide (Aβ1–42 catalog number A-1002–2; Aβ-scrambled A-1004–2) as described previously [57]. First, the peptides were resuspended in 500 μL of HFIP (catalog number AC445820100; Fisher Scientific) and left to dry overnight under a ventilated hood. The next day the samples were spun in a SpeedVac until dry and stored desiccated at −20°C until use. Prior to using, the solution was sonicated for 5 minutes and centrifuged at 1400 x g for 5 minutes.

### 3.5 AD Drugs.

Several classes of drugs used in the treatment of AD were used in this study, including Rolipram (Cayman Chemicals, Cat. # 10011132), Saractinib (Cayman Chemicals, Cat. # 379231-04-6), Memantine (Cayman Chemicals, Cat # 14184)and Donepezil (Sigma, Cat. # D6821). Memantine is an uncompetitive N-methyl-D-aspartate (NMDA) receptor antagonist that binds to the receptor and blocks the binding of glutamate and thus cell overexcitotoxicity caused by glutamate [58]. Donepezil is an FDA-approved drug for AD, it is an acetylcholinesterase inhibitor which prevents the breakdown of acetylcholine in the synapse by the acetylcholinesterase enzyme [59, 60]. Saracatinib inhibits the action of Fyn, a member of the tyrosine kinase family that phosphorylates other proteins including tau and NMDAR, resulting in tau hyperphosporylation and synaptoxicity/or cells excitotoxicity from NMDAR activation [61–64]. Rolipram is a selective phosphodiesterase-4 (PDE4) inhibitor that helps to restore cAMP level which is affected (i.e., reduced) in AD as a reslust of adenylate cyclase (synthesizes cAMP) inactivation by Aβ42 peptides [65]. All drug stocks were prepared by reconstitution in 1X PBS.

### 3.6 Drug Treatment of Cortical Neurons:

Neuronal cultures were treated for patch clamp via half medium exchange containing either Aβscr or Aβ42 oligomers with or without AD drugs 24 hours prior to testing. For MEA analysis, cells were fed at 24 hours prior to treatment by performing a half medium change to prepare the cells for the intensive activity testing the next day. Upon testing time, following the 5 min baseline testing, LTP induction and 5 min recording right after the induction, the cells were either dosed with a final concentration of 5 μM Aβscr, 5 μM of Aβ1–42 alone, or 5 μM of Aβ1–42 plus drug (either 10 nM of saractinib, 1 μM of donepezil, or 5 μM of memantine, 1 μM of rolipram). To examine the neurotoxic effects of Aβ42 oligomers on cell electrophysiological function, the cells or cultures were tested at 1 hour (MEAs) following treatment by conducting another 5 min recording. At the end of the activity testing, a dose of 1mM Lidocaine) was always applied to silence all the neural activity so as to confirm the biological source of the recorded signals. Those signals that were not abolished by Lidocane would be considered as noise and will be excluded from MEA analysis.

### 3.7 Immunocytochemistry and Confocal Microscopy:

To analyze the cells for gene expression of the drug targets, the cells were fixed in 4% paraformaldehyde (PFA), followed by cell permeabilization and incubation in primary and secondary antibodies solutions (diluted in BSA/NGS/T20 buffer) for each specific marker. Following antibodies staining, the cells were counterstained with DAPI (4’,6-diamidino-2-phenylindole) and mounted on glass slides for analysis. The cells were imaged using a confocal microscopy (Zeiss, Axioskop 2, Germany). The following primary antibodies (at 1/1000 dilution) were used: Rabbit Anti-Microtubule-Associated Protein 2 (Millipore, Cat. #: AB5622), Mouse Anti-MAP2 (Abcam, Cat. #: ab11257), Mouse Anti-Fyn (ThermoFisher, Cat. #: MA1–15865), Mouse Anti-NMDAR2B (ThermoFisher, Cat. #: MA1–2014), Mouse Anti-Acetylcholinesterase (Abcam, Cat. #: ab2803), Mouse Anti- muscarinic Acetylcholine Receptor (Abcam, Cat. #: ab90805), Rabbit Anti-nicotinic Acetylcholine Receptor (Abcam, Cat. #: ab221868), Rabbit Anti-Phosphodiesterase Type 4 (Abcam, Cat. #: ab14628). The secondary antibodies used: Alexa-Fluor 488 goat anti-rabbit (ThermoFisher, Cat. # A11008), AlexFluor 488 Goat anti-mouse (ThermoFisher Cat. #: A11001), AlexFluor 568 Goat anti-Rabbit (ThermoFisher Cat. #: A11036), and AlexFluor 568 Goat anti-mouse (ThermoFisher Cat. #: A11004).

### 3.8 Patch-Clamp Electrophysiology Recording of Cortical Neurons:

To measure individual neuronal activity, whole cell patch-clamp recordings were taken using a Zeiss, upright microscope (Axioscope, FS2, Carl Zeiss, Germany) equiped with a multiclamp 700B amplifier and an intracellular solution consisting of 140 mM K-gluconate, 4 mM NaCl, 0.5 mM CaCl2, 1 mM MgCl2, 1 mM EGTA, 5 mM HEPES Acid, 5 mM HEPES base and 5 mM Na2ATP as described previously [53]. Depolarization-evoked inward and outward currents were examined in voltage-clamp mode while induced action potentials (APs) were recorded in current-clamp mode. Sucessive analysis of the data were carried out using pClamp 10 software (Axon Instrument, Foster City, CA, USA) followed by quantification using Microsoft Excel and GraphPad Prism.

### 3.10 Long-Term Potentiation Induction on MEAs>:

To induce LTP on cortical neurons cultured on MEAs, a high frequency stimulation (HFS) protocol was used as described previously [53]. Test stimuli were delivered to all the electrodes in the form of 80 pulses at 100Hz. The evoked response or induced cell activity was then analyzed using Anaconda with Python (i.e., Monday.com) software. The waveforms (e.g., action potential spikes and frequency) were thresholded at −5 standard deviations from the noise and high-pass filtered at 100Hz. Any electrodes with firing frequency post-stimulation filess than or equal to baseline levels were excluded from the data analysis.

### 3.11 Analysis of Cortical Neuron Firing Activity on MEAs:

The cellular activity of the cells was measured extracellularly on MEAs as described previously [35, 53, 66]. The cells were plated directely onto the MEA chips in housing and maintained in culture for 28–35 days before LTP experimentswith Aβ oligomers and AD drugs. Prior to treatment, spontaneous activity (baseline) of the neurons was recorded (for 5 minutes) followed immediately by electrical stimulation (LTP induction) and another 5 minutes recording post-stimulation. Immediately following LTP induction and recording, the cultures were treated with Aβ oligomers and/or Aβ oligomers AD drugs and incubated for 1 hour at 37°C and 5% CO2. Next, the neuronal firing potential (e.g., firing frequency) was once again recorded for 5 minutes before analysis. The data was analyzed using Anaconda with Python software.

### 3.12 Statistical Analysis:

Comparison of the mean of at least three or more replicates and more than 15 electrodes between groups was performed. For computational analyses, Microsoft Excel software and GraphPad Prism were used. Student t-tests were used for statistical comparison analysis between two experimental groups, whereas one-way analysis of variance (ANOVA) with Tukey’s post hoc test was used for multiple experimental groups. The SEM was used with statistical significance taken at p ≤ .05.

## Data Availability

Data is provided within the manuscript or supplementary information files. The datasets are available from the corresponding author on reasonable request.
